# Guidance for Building Hospital at Home: Qualitative Descriptive Thematic Analysis of a Pan-Canadian Community Participatory Workshop Series

**DOI:** 10.2196/88921

**Published:** 2026-07-14

**Authors:** Tianna Magel, Quynh Ho, Maria Montenegro, Megan MacPherson, Clare L Ardern

**Affiliations:** 1 Centre for Aging SMART, The University of British Columbia, Vancouver, BC, Canada; 2 Department of Physical Therapy, Faculty of Medicine, The University of British Columbia, Surrey, BC, Canada; 3 Department of Virtual Health, Fraser Health, 13450 – 102nd Avenue, Surrey, BC, V3T 0H1, Canada, 1 604-587-4600; 4 Department of Occupational Science and Occupational Therapy, The University of British Columbia, 13737 96 Avenue, Surrey, BC, V3V 0C6, Canada

**Keywords:** digital health, delivery of health care, remote patient monitoring, telemedicine, patient-centered care, primary health care, qualitative research, home care services, hospital based

## Abstract

**Background:**

Virtual health care models, such as *Hospital at Home* (HaH), are an alternative to in-person care and allow hospitals to expand care capacity and delivery without the need for additional brick-and-mortar structures. While generally well received, there is an overall lack of awareness among those receiving and giving care about what HaH is and what it does, and uncertainty about the conditions needed to implement HaH in a safe, sustainable, and equitable way.

**Objective:**

In this descriptive qualitative study, we aimed to synthesize key interest holder–generated perspectives about the implementation, sustainability, and equity of HaH models in Canada using data generated through a national participatory workshop series.

**Methods:**

Health care providers, patients, caregivers, and hospital administrators were recruited to participate in the workshop series through social media, professional, and community networks. Five 60-minute virtual workshops were held from January to June 2025 using a “Lunch and Learn” format and *Liberating Structures* techniques. The workshops each comprised 2 parts: speaker presentations followed by participant group discussions. Workshop presentations, along with 3 written observer reports (from a patient partner, an academic, and a clinician), and workshop attendee polling responses, were collected and analyzed. Descriptive thematic analysis was used to construct key themes salient to the data.

**Results:**

Three themes were constructed from the data: (1) making HaH work for health care systems, (2) making HaH work for its people, and (3) making HaH better now and in the future. Participants reported generally positive outcomes and high satisfaction with HaH programs in Canada. Participants highlighted the need for clear communication and collaboration across care teams, technology support for staff, managing health care provider and caregiver workloads, and ensuring access for rural and remote communities. There is a need to better understand the economic sustainability of the HaH program and to study and share outcomes from HaH models to help others and refine the model.

**Conclusions:**

This study provides insights into how the HaH virtual care model is perceived by health care providers, patients, caregivers, and hospital administrators in Canada. Our findings highlight the importance of equity, communication, care workloads, and fiscal sustainability in supporting HaH models.

## Introduction

### Background

Global health systems and hospitals are operating at or above capacity, battling overcrowding, limited hospital beds, extended wait times, and provider burnout [1-3]. In North America, costs for providing health care have increased substantially in the face of worsening patient health outcomes [4,5]. These health system pressures have been exacerbated since the COVID-19 pandemic, leaving health care systems struggling to meet current demands. *Hospital at Home* (HaH) models can expand care capacity without the need for additional physical health infrastructure and have been touted as a way of reducing system-level burdens placed on hospitals [6].

HaH was first introduced by Johns Hopkins in the mid-1990s to manage patients who declined hospital stays or who were at risk of contracting an infection in the hospital [7]. As health systems and technologies have evolved, global interest in and demand for HaH have expanded, with models being adopted in several countries, including Canada, the United States, Australia, the United Kingdom, and parts of Europe. HaH offers a combination of in-person visits and remote monitoring by health care providers and is tailored to the care landscapes and insurance practices of local hospitals [8-10].

While there are several models for designing and implementing HaH, most follow an extended nursing care model, where nurses (or paramedics) perform daily home visits and an overseeing physician provides daily assessments, either remotely or in person [4]. The program offers hospital-level treatments, monitoring, and diagnostic services in the relative comfort of the patient’s home [4,10-12]. When receiving care in an HaH model, patients often have shorter treatment durations, lower hospital readmission rates, and better clinical outcomes than those receiving in-hospital treatment [7,13]. HaH programs are considered safe and effective alternatives to inpatient care. They are cost-effective and generally well accepted by patients [13-15].

### Global Evidence on HaH Models

Although international evidence supporting HaH is growing, the literature remains heavily concentrated in a small number of countries, particularly the United States and the United Kingdom, whose health system structures differ from Canada’s. A recent systematic review identified 69 HaH programs described in comparative effectiveness research, of which more than half were based in the United States and the United Kingdom [16]. Most research exploring patient and caregiver perceptions of HaH originated from these 2 regions, with only a single Canadian study identified [17].

As HaH implementation is shaped by the policy environment, funding mechanisms, and workforce structures of the host country, country-specific data are required to guide tailored implementation. The United States operates within an insurance-based, market-driven system where HaH expansion has been incentivized through reimbursement reforms and private sector partnerships. The UK’s National Health Service, in contrast, is a centrally organized, publicly funded system that has integrated HaH under national strategies for community-based and intermediate care, supported by standardized commissioning and performance frameworks. Health care in Canada is publicly funded and provincially administered, with no centralized national structure for acute care innovation. There is a patchwork of community health resources, home care services, and digital infrastructure across provinces and territories. This underscores the need for context-specific evidence that accounts for Canada’s decentralized governance, mixed urban-rural service distribution, and integration challenges between hospital and community care sectors.

### The Canadian Context

Early Canadian efforts at implementing HaH in British Columbia and Alberta (eg, through Island Health’s HaH initiative and Calgary’s Complex Care Hub, respectively) have delivered encouraging outcomes, including high patient satisfaction, reduced readmissions, and cost-effectiveness when care was provided early [18]. Despite the potential, Canada has been slow to adopt HaH, with limited national coordination or shared learning across settings. In Canada, achieving a scalable, equitable HaH model will require deliberate system-level design, adequate resourcing, and alignment across sectors [18,19].

Bringing together patients, caregivers, health care professionals, researchers, administrators, and policy leaders can help surface diverse perspectives on HaH design, delivery, and evaluation. However, structured opportunities for such exchange have been limited in Canada, and few mechanisms exist to support collective reflection on what is required to implement HaH in a safe, equitable, and sustainable way.

The aim of our study was to analyze data that were generated through a 5-part workshop series, where we engaged interest holders in dialogue about HaH in Canada. This paper aimed to describe the design and facilitation of a workshop series, outline the use of participatory methods to engage participants, and present the findings from a qualitative analysis of participant reflections. While our work does not directly explore the issues of financial and health constraints within Canadian health care systems, it does capture these points as raised by participants.

## Methods

### Study Design

This study used a qualitative descriptive design. We analyzed data from workshop presentations, interactive group discussions, observer reflections, and audience polling conducted using the online platform Slido (Cisco Systems Inc).

### Ethical Considerations

The study received ethical approval from the Research Ethics Boards (REBs) at The University of British Columbia and Fraser Health (REB H24-03016). The procedures followed were in accordance with the ethical standards of the REBs and with the WMA Declaration of Helsinki. All participants provided written informed consent prior to participating in the workshops, and their rights to privacy and confidentiality of data and identity were protected. Participation was voluntary, and no compensation was provided to participants.

### Workshop Format and Structure

#### Overview

The Canadian Hospital at Home Working Group (HaH Working Group) is a national, community-based collaborative established in 2023 to address the limited recognition and operational integration of HaH models within Canadian acute care systems [20]. The group operates as an open community of practice of approximately 50 members, bringing together clinicians, researchers, and operational leaders from across Canada to advance awareness, promote standardization, and facilitate knowledge exchange to support the broader adoption of this evidence-based approach. For our study, we partnered with the HaH Working Group, drawing on its established membership and expertise to inform the design, delivery, and hosting of the workshop series. The HaH Working Group members contributed to this project by shaping workshop themes through monthly meetings and asynchronous feedback, serving as speakers, and actively promoting the workshops within their networks.

A series of 5 virtual workshops, using a “Lunch and Learn” format, was held between January and June 2025. The workshops were designed as a participatory engagement activity to explore (1) implementation and safety, (2) patient and provider experiences, and (3) evaluation and communication of HaH models across Canada. We incorporated approaches to foster cross-sector knowledge exchange and engagement and to inform future HaH initiatives through structured reflection and collaborative learning.

We used *Liberating Structures*, a set of participatory facilitation methods, to promote inclusive conversation, creative thinking, and equal contribution from participants [21]. Liberating Structures are well suited to complex, multiperspective discussions where traditional top-down approaches may inhibit engagement. Liberating Structures give all members a chance to express their opinions, fostering engagement across groups of all sizes [22]. Specifically, Liberating Structures enlists 33 different approaches, such as 25/10 Crowdsourcing, an approach to rapidly generate actionable ideas, and 1-2-4-All, which engages all members of a group regardless of size, to encourage engagement from all participants [21]. Using this approach, we designed, delivered, and studied a national series of virtual workshops to engage health system participants (patients, caregivers, providers, and administrators) in conversations about implementing HaH in Canada.

The workshops were designed as a participatory engagement initiative to convene diverse interest holders and facilitate dialogue on HaH in Canada. While these workshops aimed to support knowledge exchange and inclusive discussion, these engagement-oriented objectives were not evaluated as study outcomes. Each workshop ran for 60 minutes and was delivered via the videoconferencing platform Zoom (Zoom Communications Inc). Each workshop was structured in two parts: (1) a short set of presentations or storytelling to provide context and provoke reflection, followed by (2) an interactive group dialogue using Liberating Structures (see Table 1 for a summary of each workshop and the Liberating Structures used) [21], which emphasized each participant having an equal voice to foster creativity and positive dialogue [22]. Liberating Structures has been used successfully in a variety of situations with various populations [23-25]. Workshop facilitators ensured psychological safety through ground rules and clear guidance, and virtual breakout rooms were used for small group exchanges. Participants were always reminded of their right to opt out or withdraw at any time.

**Table 1.** Overview of workshop sessions and Liberating Structures used.

Workshop	Description	Liberating Structures used
Introduction to HaH^a^	Introduced the foundational concepts of HaH, with speakers presenting different models of care delivery and exploring what a patient-centered experience can look like in HaH settings.	1-2-4-all (participants reflected individually, in pairs, in small groups of 4, then with the full group)
Safety in HaH	Explored how patients and providers defined and ensured safety within HaH programs, including examples of safety innovations, outcomes data, and improvement initiatives.	W**^3^** (*What? So What? Now What?*)
Patient and provider experience	Examined the lived experience of patients and health care professionals involved in HaH, including themes of satisfaction, communication, and continuity of care.	W**^3^** (*What? So What? Now What?*)
Evaluation practices in HaH	Addressed evaluation strategies used by HaH units, highlighting what matters most to participants in assessing impact and how programs are currently measuring outcomes.	25/10 Crowd Sourcing (participants generated ideas and rated each idea; 25 points were awarded for the highest score)
Communication in HaH	Explored best practices for interprofessional communication and information flow in the HaH context, using case scenarios to identify system gaps and opportunities to improve.	Wise Crowds (participants collaboratively consulted on a real-world challenge)

a HaH: Hospital at Home.

Three designated observers (a patient partner, an academic researcher, and an early-career clinician with firsthand experience and knowledge of the health care system) were recruited using purposive sampling and either attended all 5 workshops or reviewed the recordings. Observers assumed a reflective and analytic role that was distinct from that of workshop participants. The observers did not engage in discussions or activities. Instead, they independently documented themes, patterns, and implications across workshops. No strict guidelines were given to observers when developing their reports. Observers were instructed to submit a written reflection highlighting key takeaways, how the content of the workshops connected to their area of expertise, whether they had gained any new insights or knowledge about HaH, and any future applications of what they had learned during the workshops (Multimedia Appendix 1). By systematically documenting reflections without influencing discussion, observers contributed an additional layer of interpretation that enhanced reflexivity and methodological rigor. This approach was informed by participatory conference models in which designated “witnesses” are invited to attend, listen, and synthesize recurring themes, allowing for the integration of diverse, external perspectives that extend beyond individual participant contributions and enhance the interpretive depth of the findings [26]. Observers received an honorarium—the amount was informed by guidance from the Canadian Institutes of Health Research [27].

#### Workshop 1: Introduction to HaH

The first workshop introduced foundational concepts of HaH and examples of patient experience from different public sector health organizations and research institutions. The speakers represented Alberta Health Services, the University of Calgary, and the Integrated University Health and Social Services Centre, West-Central Montreal, Quebec.

In the discussion segment, we used the *1-2-4-All* Liberating Structure to invite participants to reflect individually, then in pairs, then in small groups of 4, before reconvening to share insights with the full group. The discussion prompt was as follows: *What opportunities and challenges do you see for Hospital at Home in your context?* To guide participant thinking, we posed two questions: (1) *What would make you interested in receiving care at HaH?* and (2) *What questions or concerns do you have about HaH?* Participants were supported to assess these questions themselves without the presenters or facilitators imposing perspectives. Participants discussed the questions in increasingly larger groups and submitted their written key takeaways using the “Q&A” feature of Slido. Written responses were submitted without names or identifiers and were not linkable to participants, rendering them anonymous at the point of collection.

#### Workshop 2: Safety in HaH

This session explored how safety was conceptualized and operationalized in HaH settings, drawing on the perspectives of providers and patients. Presentations were delivered by researchers from The University of British Columbia’s Digital Emergency Medicine group and Calgary-based providers from Alberta Health Services.

The session used the *W³ (What? So What? Now What?*) Liberating Structure as a scaffold for reflective and action-oriented dialogue. Participants first reflected individually before joining breakout groups of 3 to 4 people for a facilitated discussion. Guiding questions were as follows: (1) *What key components of safety within the HaH model stood out to you? Why is this important?* and (2) *What improvements could be implemented to ensure patient safety within HaH?* The breakout group conversations were guided by simple ground rules to ensure respectful and inclusive engagement. After the breakout sessions, participants returned to the main room and submitted anonymous reflections using an online question and answer (Q&A).

#### Workshop 3: Patient and Provider Experience in HaH

Focusing on feedback and lived experiences, the third workshop featured presentations from Fraser Health (British Columbia), Calgary Zone Patient and Family Centered Care, and Alberta Health Services. The speakers shared qualitative insights and patient stories from their HaH programs.

The discussion activity again used *W³ (What? So What? Now What?*) to prompt reflective and generative dialogue. Guiding questions were as follows: (1) *Was the feedback received from patients and providers different from what you expected?* and (2) *How has your experience differed?* After a brief individual reflection, participants joined breakout rooms for small group discussion and submitted their thoughts anonymously through an online Q&A.

#### Workshop 4: Evaluation Practices in HaH

The fourth session addressed methods for evaluating HaH services. Presentations were given by Fraser Health and the Integrated University Health and Social Services Centre, West Island (Quebec). The workshop used a modified version of the *25/10 Crowdsourcing* Liberating Structure to generate and prioritize ideas. Participants were first asked to reflect individually, then joined breakout groups of 3 to 4 people for a facilitated discussion. The discussion question was as follows: *If you had unlimited resources, what key outcomes or impacts would you prioritize evaluating in Hospital at Home and why?* Without influence from presenters or facilitators, the participants discussed this question among themselves. After reconvening, participants submitted their responses through an online Q&A and were invited to vote on other responses to create a collective ranking of the top evaluation priorities.

#### Workshop 5: Communication in HaH

The final workshop focused on communication strategies and challenges in HaH programs. The speakers represented Island Health (a health authority on Vancouver Island, British Columbia) and Vancouver General Hospital (a teaching hospital in British Columbia).

The session used the *Wise Crowds* Liberating Structure, which allowed participants to collaboratively consult on a real-world challenge. The discussion scenario presented was as follows: *A patient is receiving care at home, but there are multiple healthcare providers (e.g., doctors, nurses, therapists) involved. The communication between the providers is inconsistent, leading to confusion over medication schedules, care plans, and treatment adjustments. What protocols can be put in place to streamline communication and reduce confusion?*

In small breakout groups, participants acted as “consultants” to propose strategies for improving interprofessional communication in HaH. The ideas were captured using an online poll and Q&A.

### Recruitment

The workshops were open to the public. Workshop participants were recruited online via snowball and social sampling through the Canada Health Infoway Canadian Hospital at Home Community of Practice [20,28,29]. Recruitment materials were also posted on internal chats and event boards and distributed through email and LinkedIn to external health organization partners and networks of the HaH Working Group, Fraser Health, and The University of British Columbia. Participation was voluntary, and attendees were asked to indicate their primary role and organization when they registered and joined 1 or more sessions based on their interest and availability. Before each discussion, participants were reminded of the voluntary nature of the activity and their right to withdraw at any time.

The observers and workshop presenters were recruited through Healthcare Excellence Canada and Fraser Health using purposive sampling to ensure we recruited individuals with diverse knowledge and experience of the HaH model in Canada [30].

### Data Management and Analysis

Three types of qualitative data were included in the analysis:

1. Presentation content: Speaker presentations were audio recorded and video recorded. The presentations shared data on existing HaH models and provided contextual and descriptive information. Presentations were coded and analyzed as a data source to enrich interpretation; we explored convergence and divergence across data sources. Presentation content was not used to validate, privilege, or discount workshop or observer perspectives, nor to support reflection or interpretation more than other data sources but rather to support interpretive depth and reflexive analysis. This approach allowed the research team to identify areas of alignment and tension without constraining insights or compromising the Liberating Structures facilitation approach. Patient experiences were described through a combination of provider-reported accounts, observer reflections, patient-reported data presented within the workshops, and workshop participant responses.
2. Observer reports: Three observers independently documented written qualitative reflections for each workshop in the series. Reflections focused on session content, emergent themes, and implications from the lens of the observer’s domain expertise.
3. Workshop participant contributions (polling and Q&A responses): Participants engaged in interactive discussions using Liberating Structures and submitted individual or group reflections via the electronic polling platform Slido.

### Analytical Approach

Data were analyzed using reflexive thematic analysis following Braun and Clarke’s 6-phase framework [31,32]. This approach emphasizes iterative coding, reflexive interpretation, and the development of themes grounded in the data. All data sources (workshop recordings, presentation transcripts, polling and Q&A responses, and observer reports) were first reviewed in full by 2 researchers (TM and QH) to support familiarization. An initial round of open coding was then conducted independently on a subset of the data to identify meaningful data excerpts relevant to the research aim.

Preliminary codes generated during this stage were compared and discussed to develop a shared coding framework. This framework comprised the following:

- Deductive codes, which were informed by the domains reflected in workshop topics (eg, safety, communication, and evaluation). Deductive codes were used as sensitizing concepts to support early data organization.
- Inductive codes, which were generated directly from the data to capture concepts that were not represented in the initial structure.

The coding framework was applied to the full dataset and iteratively refined through ongoing coding, discussion, and comparison. Data were not constrained to preexisting categories, and new codes were added, and existing codes were revised, as needed, to reflect the breadth and complexity of the dataset.

Coding was conducted across and within workshop datasets. Initially, data from each workshop were coded to capture context-specific patterns. Subsequently, codes were compared across workshops to identify recurring patterns and areas of conceptual overlap. Related codes were grouped into broader categories, which were further developed into themes that spanned multiple workshops. Themes were generated through an iterative process of reviewing coded data, developing candidate themes, and refining themes to ensure internal coherence and distinction between the themes.

Throughout the analysis, the research team engaged in regular discussions to reflect on coding decisions, resolve differences in interpretation, and refine the developing thematic structure. This process supported analytic rigor and reflexivity.

## Results

### Overview

Ninety-one unique participants (excluding facilitators, speakers, and observers) attended the workshop series (Table 2). Forty-one (45%) were from British Columbia, 15 (17%) from Ontario, 9 (10%) from Quebec, 8 (9%) from Alberta, 5 (6%) from Nova Scotia, 2 (2%) from Saskatchewan, and 2 (2%) from New Brunswick. Among all attendees, 52 (57%) held administrative or management positions at the time of the workshops (eg, consultant, program coordinator, manager, and director), 24 (26%) served in clinical roles (eg, physician, nurse, resident, and clinical leader), 4 (4%) were students, and 1 (1%) was a patient.

**Figure 1.**
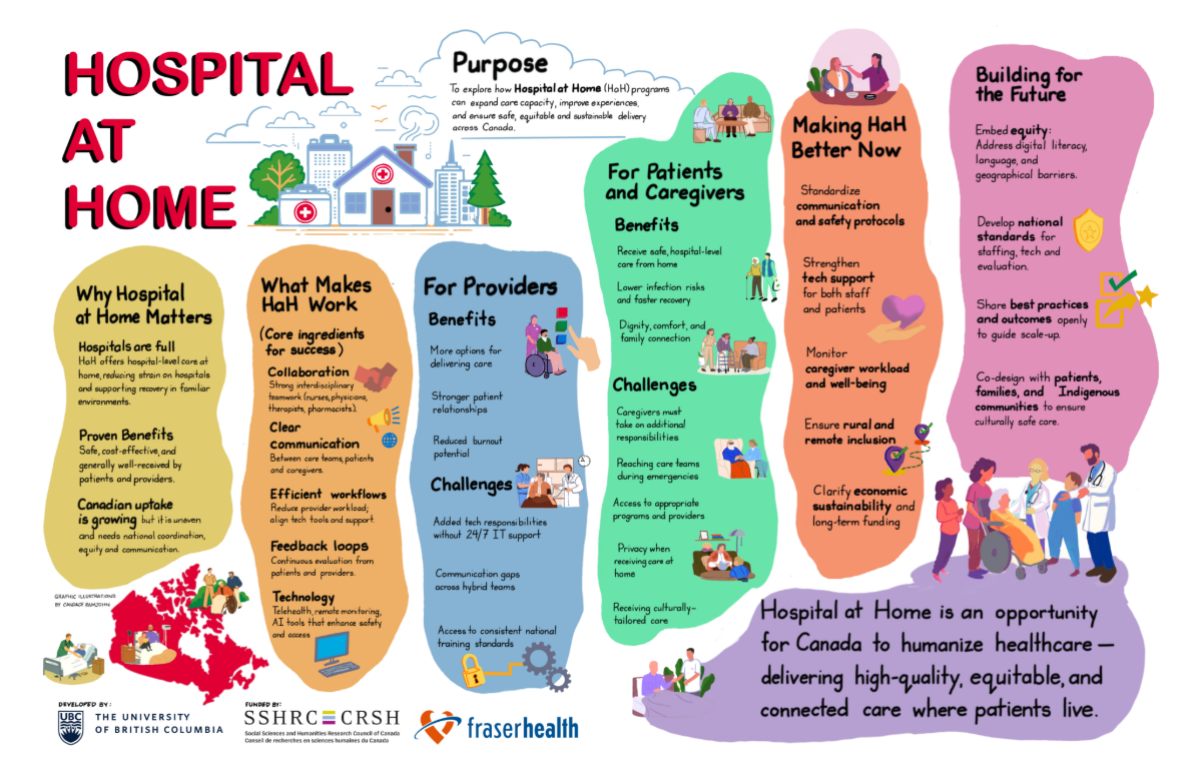
Hospital at Home (HaH) summary for knowledge users (ie, patients, clinicians, and the public) demonstrating themes and subthemes related to how HaH works; the benefits and challenges for patients, caregivers, and providers; and building for the future.

We constructed three major themes from the data: (1) making HaH work for health care systems, (2) making HaH work for its people, and (3) making HaH better now and in the future (Figure 1).

**Table 2.** Hospital at Home workshop 1‐5 participants demographic data (N=91)^a^.

Characteristics	Participants, n (%)	Workshop 1: introduction (n=40), n (%)	Workshop 2: safety (n=34), n (%)	Workshop 3: patient and provider (n=37), n (%)	Workshop 4: evaluation (n=25), n (%)	Workshop 5: communication (n=17), n (%)
Location						
British Columbia	41(45)	22 (55)	17 (50)	17 (46)	12 (48)	7 (41)
Alberta	8 (9)	—^b^	1 (3)	5 (14)	1 (4)	1 (6)
Saskatchewan	2 (2)	—	1 (3)	2 (5)	1 (4)	—
Ontario	15 (17)	11 (28)	6 (18)	4 (11)	3 (12)	2 (12)
Quebec	9 (10)	3 (8)	4 (12)	5 (14)	2 (8)	—
Nova Scotia	5 (6)	—	—	—	—	5 (29)
New Brunswick	2 (2)	2 (5)	2 (6)	—	1 (4)	1 (6)
Unknown	9 (10)	2 (5)	3 (9)	4 (11)	5 (20)	1 (6)
Role						
Administrative	52 (57)	26 (65)	22 (65)	24 (65)	11 (44)	11 (65)
Clinical	24 (26)	9 (23)	9 (26)	6 (16)	6 (24)	3 (12)
Student	4 (4)	3 (8)	1 (3)	2 (5)	1 (4)	1 (6)
Patient	1 (1)	—	—	—	—	1 (6)
Unknown	10 (11)	2 (5)	2 (6)	5 (14)	7 (28)	1 (6)

a The total number of participants does not reflect individuals who attended more than one workshop.

b Not applicable.

### Theme 1: Making HaH Work for Health Care Systems

#### Collaboration and Communication

The delivery of around-the-clock HaH care required a high degree of coordination and meaningful collaboration across care teams and subspecialties. This was noted as a “pretty important...prerequisite” (workshop 2 participant) of the program and required care teams to “stay in close touch with each other” (workshop 5 participant):

*[S]takeholder collaboration is really important between departments*.

[Workshop 2 participant]

Clear communication was also seen as fundamental to delivering “safe, smooth, and effective” (workshop 2 participant) care and was instrumental to the success of HaH. Patients were noted as wanting “clear communication and easy access to their health care team” (workshop 2 participant) for safety and for managing uncertainty. However, communication was also viewed as complex and an area for improvement. Some subspecialty providers struggled with knowing how to reach the “patient or the Hospital at Home team” (workshop 5 participant), creating barriers to scheduling procedures and providing the best care. HaH required clear communication to ensure care providers from interdisciplinary departments were aware of treatment changes or developments. Differing understandings of what HaH is across subspecialties were also seen as barriers to buy-in and referral to the program:

*By focusing [on] collaboration, clear communication, support, responsiveness, learning from real experience, we can better ensure hospital at home continues to improve patient care and healthcare system efficiency*.

[Workshop 2 participant]

Efficient workflows were also highlighted as an important component of coordinating between different hospital departments and accommodating hybrid care. This was important for mitigating provider perceptions that HaH increased “demand on [provider] resources” (workshop 2 participant) or workload. Some strategies adopted to manage workflow challenges included monthly meetings with leadership and regular community-based team huddles and continuous evaluation of daily resources to ensure workflows were fit-for-purpose:

*Providers spoke about the need for streamlined workflows, real-time monitoring, and patient-reported outcomes to ensure quality and accountability*.

[Observer 2]

The “hub-and-spoke” (workshop 5 participant) model, in which physicians provided remote care to patients via virtual platforms and coordinated with the community-based nursing teams (or paramedics) who provided daily in-person patient visits, was highlighted. The *hub-and-spoke* model was noted as helping to remove the barriers of requiring physicians to make at-home visits.

#### Concerns About Implementation

Safety concerns were centered around treating at a distance and ensuring appropriate access to and communication with care teams. Medication was also a focus of safety, which included the administration and titration of patient medications from a distance and patient noncompliance. Accessibility of care teams in rural and remote settings was raised as a concern, as were secure environments and “contingency plans” (observer 2) in the event of a power failure or an emergency:

*[A]dditional measures like medication adherence, safety incidents, and care coordination effectiveness should also be examined to link to clinical and care delivery outcomes*.

[Observer 1]

*Clear communication around accessing support when needed. What do I do if I cannot get a hold of anyone?*

[Workshop 1 participant]

The need to better understand the “longer-term safety and health outcomes” (observer 1) of HaH was also highlighted. There were questions about the efficacy of HaH and how “results compare[d] to those of patients admitted to brick-and-mortar units only” (workshop 4 participant). The admission process and whether patients would have to wait to be admitted to HaH again if they required acute care after discharge were also questioned:

*How do HAH outcomes compare to other Remote Patient Monitoring (RPM) models of care?*

[Workshop 4 participant]

Concerns were raised about care providers navigating new technology and ensuring that providers were “sufficiently competent to provide care, and support patients' navigation” (observer 1). Further training was noted as being needed to “improve [the] technical tools and skills of staff” (workshop 3 participant). Integration of differing technologies was seen as a way to offer diverse types of virtual care and create “additional capacity and resilience” (workshop 1 participant) for organizations. Integrating technologies, including artificial intelligence, was seen as a way to potentially streamline interactions with patients, making clinical encounters more efficient.

### Theme 2: Making HaH Work for Its People

#### Providers and Staff

A high degree of support for and satisfaction with the HaH program was noted from the provider data. The quality of care provided by HaH was seen as “excellent” (workshop 3 participant), offering flexibility and an alternative to typical shift work and a way to avoid “burnout” (observer 3). The HaH program minimized hassle and allowed providers to effectively monitor patients:

*[...] from a provider perspective, hassle was minimized. [...]there were efficient coordination systems in place, allowing [providers] to monitor patients effectively without excessive administrative burdens*.

[Workshop 2 participant]

On the other hand, HaH models may also add more “complexity and uncertainty” (workshop 5 participant), require more planning and “longer encounter times” (observer 1) than traditional health care models where patients are admitted to the hospital. HaH could also place a greater burden on staff, with many “bearing the tech[nology] support burden” (observer 1). Additionally, increased staff turnover was flagged as a concern, noting the need for a “longitudinal understanding” (observer 1) of staffing needs for health care providers to ensure HaH is successful:

*A new healthcare model cannot be sustainable if the providers delivering the care are overworked and/or under-equipped*.

[Observer 3]

Providers were generally seen as having to take on IT support and troubleshooting responsibilities in the absence of a 24/7 technology support team. Concerns were raised about placing the brunt of troubleshooting on staff and increasing provider workloads. High levels of provider on-the-job learning and the addition of “a lot more complexities in a virtual unit” (workshop 3 participant) were noted:

*Sites with positive experience with patients having tech[nology] support work out well for them. The counter to that is it’s our staff. It’s our clinical bodies that are essentially having to make sure that patients don't feel the frustrations about the technology, but they're kind of wearing it themselves*.

[Workshop 3 participant]

#### Patients and Caregivers

Across data sources, the care delivered through HaH was perceived to be of high quality, with some noting that it was described as “better than or the same as inpatient level care” (workshop 2 participant). Perceived benefits associated with receiving care at home, including the potential of being at home to “reduce recovery time” (workshop 2 participant) and improved health-related quality of life, as discussed during workshop presentations and group dialogue, were also identified. Being at home was described as allowing patients to “be close to [their] family” (workshop 1 participant) and receive care in a familiar and comfortable environment. HaH was also characterized as less disruptive and as supporting care with “dignity” (observer 2) while reducing exposure to hospital-based risks such as infection:

*Receiving care in the comfort of my own home. - Reduced risk of being infected by viruses, etc. in the hospital environment*.

[Workshop 1 participant]

Technology was generally discussed positively, with confidence in its use and in the security of personal health information reported. At the same time, the need for clear protocols in the event of an emergency, including concerns about “struggling to reach someone during critical moments” (workshop 2 participant), was emphasized. The need for caregiver supports to be built into future HaH models was discussed, with potential compensation for caregivers who were absorbing additional roles. It was noted that the HaH program could heap additional stress on caregivers and family members who “take on extra roles in [providing] care” (workshop 1 participant) and who otherwise would have had “a break” (workshop 2 participant) while their loved one was in hospital:

*[...] the acknowledgement of how HaH can increase responsibilities of family members. This feedback is important to keep in mind with this shift in healthcare; HaH can empower families, but it requires thorough preparation and ongoing support to avoid caregiver burnout*.

[Observer 3]

Patient-centered care and patients being “involved in their care decisions” while being “treated with dignity and respect” (workshop 3 participant) were highlighted as important pillars of HaH. These included patients feeling heard, “meeting expectation[s], ... reliability, [and] ensuring [that] what was promised in hospital at home was delivered” (workshop 2 participant). Providing patient-centered care also extended to providers and ensuring their well-being when delivering care within the HaH program:

*This gave a deeper appreciation for how patient-centered the Hospital at Home model truly is*.

[Observer 3]

### Theme 3: Making HaH Better Now and in the Future

#### Now

Evaluation and interest holder feedback were considered critical components in continuously improving the HaH program and ensuring patients feel safe and heard. The need for “rapid cycle feedback” (workshop 2 participant) that included feedback on areas for improvement and safety from health care providers and input from patients and caregivers was noted.

Systematic approaches to evaluating HaH and a need for national standards (eg, charting methods, language, and evaluation outcomes) to ensure consistency across interdisciplinary professions were stressed as important requirements for HaH. Standards for technological training were also flagged, highlighting the need for more clarification on “how clinicians [were] trained” (observer 1) and how training was received in HaH settings:

*Subspecialties can sometimes have a different idea of what Hospital at Home is and what it can do... it’s interesting that different people will have different understandings of the same thing*.

[Workshop 5 participant]

More information about how the HaH model aligned with “health system priorities” (workshop 4 participant) and the total cost was important in assessing the return on investment for the program. In the current health care budget crisis, budget limitations were flagged as an important consideration in the long-term “economic sustainability” (observer 1) and “fiscal cost” (workshop 1 participant) of HaH. How organizations with limited budgets might implement similar models and whether resource allocation was being considered for rural and remote communities were also questioned.

*Ensuring that there is consideration of the efficiency and fiscal cost of the model. What patient populations can be looked after as efficiently or more efficiently in HaH compared to the hospital?*

[Workshop 1 participant]

#### In the Future

More information was desired on how HaH could be adapted to different populations. The need for equitable access (eg, transportation, medication costs, and digital literacy) for “rural and resource-limited areas” (observer 3) was also highlighted. The hybrid nature of the model was noted as resulting in a “geographic catchment area” that limited “the distance that [care teams] can be from [their] patients” (workshop 5 participant):

*One concern [...] was equitable access. While HaH could be transformative, rural and resource-limited areas may not have the access that is needed for this model to be successful*.

[Observer 3]

Health equity, access, and “who is being excluded from these programs” (observer 1) were raised as important points for future iterations of the HaH model to consider and address. Adapting HaH to suit specific populations (eg, children or older people) and to guarantee equitable access to “specialists outside the HaH program” (workshop 1 participant), as well as culture, language, and privacy in home environments of different cultures and backgrounds were seen as important:

*I think discussions around equity and access, particularly addressing the needs of patients from diverse cultural and linguistic backgrounds, need to be addressed*.

[Observer 2]

The importance of spreading awareness about HaH and sharing findings and publications to support learning or implementation was underscored. In building their own HaH model, it was helpful to build “off the work and learning of others” and “not start from scratch” (workshop 4 participant):

*It is essential that we share and publish our experiences and research so others can learn from it too*.

[Workshop 5 participant]

Future iterations and evaluations of HaH were seen as benefiting from the representation of patient and caregiver voices. Engaging patients and caregivers early and often and considering “different ways of getting people’s feedback” (workshop 4 participant), including using accessible language, was seen as important for ensuring that evaluations of HaH resonated with audiences. The importance of transparent discussions about the costs of HaH to the health system when considering future expansion was also noted:

*[...] patients, caregivers, and providers each see different benefits and challenges, and [...] these perspectives must all be integrated if HaH is to succeed*.

[Observer 2]

## Discussion

### Principal Findings

Our findings highlighted three main themes related to HaH models: (1) making HaH work for health care systems, (2) making HaH work for its people, and (3) making HaH better now and in the future. While HaH was viewed as a safe, patient-centered alternative to inpatient care, its success and sustainability depend on coordinated system design, clear governance, and equity-focused implementation strategies. As Canada continues to navigate increasing demand and burden on its health care system, HaH offers a model that might alleviate some pressures and ensure timely access to care. We took a community engagement approach to examining HaH programs across Canada. We conducted the workshops as a participatory engagement initiative to better understand the public perception of HaH within the Canadian health care system, to increase awareness about the program, and to identify how innovation and collaboration could expand across health care systems. We explored national HaH models from different communities and examined interest holder awareness of, and the perceived barriers to and facilitators of, implementing HaH. Aligning with previous literature, our findings highlighted the need for clear communication, hospital infrastructure, provider training, and digital literacy considerations [4,7,33]. We extend previous evidence by highlighting the need to better consider equity, caregiver and physician burdens, and embedding patient and caregiver voices when developing HaH models (Figure 1).

### Strengthening Governance and Coordination

Participants were looking for clear governance, national standards, and consistent definitions of HaH. They desired standards for HaH health care providers and models to promote consistency across programs. Developing national standards could support health care organizations to adopt HaH, without the added burden of each organization separately investing in programs and trialing various iterations of the HaH model. The existing HaH network via Canada Health Infoway could be engaged to catalyze national coordination across Canada. Learning from what others have done in the Canadian context could expedite the expansion of HaH models around the world and could enhance consistency across programs by developing Canadian guidelines for clinical governance, safety, and evaluation. An assessment of the feasibility and impact of developing a pan-Canadian HaH network is warranted in the future.

### Building Workforce Capacity

Given the emphasis on multidisciplinary collaboration and technology use, national investment in HaH-specific training, digital competencies, and interprofessional education will be essential to advancing HaH in Canada. Current HaH models could benefit from boosting provider training to support HaH-specific care and technological competency. Upfront investment in training can help mitigate downstream interruptions to workflow and on-the-job learning curves [7]. This includes investment in training support staff, such as paramedics, to fill personnel gaps and coordinate care [33]. The remote nature of HaH also requires diligent, coordinated care and clear communication to ensure providers are aligned when it comes to a patient’s treatment. Regular team check-ins can help providers coordinate care, boost timeliness, and reduce workload barriers [34]. Care teams that prioritized team communication, via team huddles or meetings, were also able to streamline workflows and reduce burden on HaH staff [34].

### Integrating Digital Infrastructure and Technology Support

Future HaH models will need to integrate appropriate training, ensure equitable digital health infrastructure, and implement user-friendly virtual platforms. Reliable technology and 24/7 support were viewed as foundational to delivering safe HaH. While comfortable with technology, patients were concerned about an overreliance on technology and the risk of technological failure during an emergency. Assessing and supporting the digital literacy of patients could be integrated into HaH models to help mitigate some of these concerns.

There is limited information on emergency response in HaH settings. Some home care programs have implemented disaster response protocols that include writing emergency plans tailored to each patient, as the patient is enrolled in the program [35,36]. As the use of remote monitoring tools expands with the growth of artificial intelligence, hospital workflows will need to plan for and provide appropriate training for patients, particularly for older adults and resource-limited populations [37-39], on how to respond to a technology failure. Health care providers reported feeling comfortable with technology but often found themselves taking on the role of IT support for patients. Regular training and integrating specialized technology support teams could help to offset the additional workload of care providers.

### Embedding Patient and Caregiver Voices

Co-designing new HaH models with patients and caregivers was considered important to ensure safety, equity, and trust, particularly as care responsibilities shift into the home. Patients and caregivers want to be involved in and provide feedback on how care is delivered. Increasing caregiver burdens have been noted as barriers to HaH uptake by patients [40]. HaH models can shift a large burden of care to caregivers, who might find themselves taking on the lion’s share of responsibility for coordinating care [41]. Participants emphasized the need for additional caregiver supports, including considering financial compensation, in mitigating the additional responsibilities and stress. While Canada offers employment insurance for family caregivers of adults, this coverage is only for a maximum of 15 weeks and is only offered to those who provide support for family members who are critically ill or in need of end-of-life care [42].

HaH is often not available to people who live alone or whose family is not in agreement about taking on caregiving responsibilities [43]. A large component of HaH’s success is in ensuring buy-in from caregivers and providers who may be more inclined to refer a patient if both patient and caregiver concerns have been addressed. Future iterations of HaH need to factor in caregiver perspectives or explore options for integrating at-home care supports.

### Addressing Equity and Access

To avoid perpetuating inequities, people who are implementing HaH in Canada must account for the country’s geography and diversity, including rural and Indigenous communities, varying digital literacy, and addressing cultural considerations. This includes explaining HaH in culturally sensitive terms and ensuring that it resonates with patients [44]. Distance from the hospital is also a barrier to providing equitable services [43]. Some researchers suggest that HaH should not be offered to those who are more than 40 km (25 miles) from an emergency department [45]. However, this approach is not feasible in rural and remote communities that may not have a local hospital.

Intermittent internet connection, adverse weather, and challenging terrain, more often seen in rural and remote locations, also create barriers to offering HaH. In the United Kingdom, remote care has been led by nursing in collaboration with volunteers from diverse communities to deliver culturally sensitive care and support for patients [46]. However, for those receiving internet support with their care, limited or no internet access after HaH has demonstrated increases in readmission and mortality [47]. Given challenges in access to family medicine and nursing in Canada, providing safe HaH in remote communities may require new and creative approaches [48,49]. Providers of HaH must engage with rural and remote communities to better understand how HaH can be adapted to meet their needs [50].

### Strengths and Limitations

A strength of this study was its participatory and multimodal approach to data collection and analysis. We analyzed data from complementary sources, engaging in national workshops, with presentations and reflection sessions, to gather a comprehensive understanding of HaH. Our study highlighted the importance of using technology to support care delivery and safety, ensuring health care providers are technologically competent, and identifying the hub-and-spoke model as advantageous for coordinating care. Our approach meant we could triangulate perspectives from providers, administrators, patients, and caregivers to explore operational and experiential dimensions of HaH beyond the academic literature. Observers systematically documented reflections without influencing the discussion, contributing an additional layer of interpretation that enhanced reflexivity and methodological rigor.

Our study design and analytic approach also strengthened the credibility and transferability of findings. The workshop format encouraged open dialogue and co-interpretation of themes, while the formal presentations and written reflections provided structured, detailed accounts of program implementation.

The limitations of our work largely stem from findings being biased toward provider and health care authority perspectives and experiences. These perspectives may have biased the discussions toward system efficiency, expansion, and cost measures. Some participants had incomplete data (ie, did not complete the “role” question on our demographic form). It is possible that these participants had important perspectives that were not fully reflected in the captured data. Workshops were delivered online to primarily urban audiences (a high proportion of whom were in British Columbia) and may not have adequately captured the barriers experienced by HaH programs offered in more rural and remote settings. Given the lack of representation from patients, caregivers, and those from rural locations, conclusions drawn from this work may be missing important perspectives and considerations for those receiving and delivering HaH care. These considerations are further explored in the *Discussion* section in relation to equity, access, and digital infrastructure. Additionally, while fostering relationship building and mutual understanding across participants was an important engagement goal of the workshops, these were features of the engagement approach that were used to support inclusive dialogue and data generation, rather than outcomes evaluated in this study.

The voices of people with low digital literacy or poor internet coverage may not be represented, and instead, the perspectives of the “early adopter” of HaH who has the income and means to engage with technology may be overrepresented. The time of day the workshops were held may also have impacted who could attend. Those with inflexible work schedules or without the luxury of taking time out for a workshop may have been unable to attend. However, given the nascent state of HaH in Canada, our results present an opportunity to start contributing to the literature on the HaH model of care.

### Conclusions

Strengthening interdisciplinary and team coordination, integrating patient and caregiver voices, addressing equity, and supporting workforce capacity and technology use were all identified as key components of successful HaH models. Our work amplifies community voices and perceptions of the HaH model in Canada. We offer pragmatic suggestions for how current HaH programs are received and how they can be improved for future expansion.

## Supplementary material

10.2196/88921 Multimedia Appendix 1 Observer instructions.

## Abbreviations

HaH: Hospital at Home
Q&A: question and answer
REB: Research Ethics Board
