# Supplementary material for: Guidance for Building Hospital at Home: Qualitative Descriptive Thematic Analysis of a Pan-Canadian Community Participatory Workshop Series
Source: J Med Internet Res. 2026 Jul 14;28:e88921. doi: 10.2196/88921 (PMC13367944; doi:10.2196/88921)
Supplement: Multimedia Appendix 1 [file jmir-v28-e88921-s001.docx]

Multimedia Appendix I. Instructions for Hospital at Home Observer reflections

**Your role**

As an observer, your role involves attending the workshops, listening to the expert speakers and participants, and reflecting on the insights shared.

Each workshop will include a discussion portion, during which participants will be divided into breakout rooms. As an observer, we ask that you refrain from participating in these discussions, but instead focus on actively listening and reflecting on the conversations.

After the series, we will ask you to submit a written reflection highlighting key takeaways and how the content connects to your own area of expertise (e.g., as an academic, health care provider, or patient). This reflection would provide an opportunity to analyze the evolving role of Hospital at Home models, reflect on how these developments impact your field or profession, and share your perspective on the challenges, opportunities, and future directions for virtual care.

There are no strict guidelines for your reflections, so we encourage you to share your own insights and personal perspectives. Below is a list of an example for how reflections can be structured to help you get started:

1. **Key Takeaways**:
   Summarize the main points or ideas discussed. Focus on what stood out to you most, whether it’s a concept, a piece of advice, or a topic that sparked new thinking.
2. **Personal Connection**:

Relate the content to your work or experience. Did anything challenge your current thinking? How do the ideas presented relate to your professional or personal journey?

1. **Insights and Learnings**:
   Share any new insights or learnings you gained from the workshop. This can be about the subject matter, the way it was presented, or the perspectives shared by the participants.
2. **Future Application**:
   Consider how you might apply what you’ve learned. Are there any actions, strategies, or ideas you plan to implement in your own work or practice?
3. **Additional Comments**:

Include any further thoughts or questions.
